# Chronic slowly progressive monoarthritis tuberculosis of the hip without systemic symptoms mimicking osteoarthritis: a case report

**DOI:** 10.1186/1757-1626-2-6457

**Published:** 2009-03-10

**Authors:** Carina Guedes Ramos, Marcelle Duarte Alves, Rodrigo Pires dos Santos, Diego Falci, Luciano Zubaran Goldani

**Affiliations:** 1 Infectious Diseases Unit, Hospital de Clínicas de Porto Alegre, Universidade Federal do Rio Grande do Sul, Ramiro Barcelos 2350, Porto Alegre, RS 90035-002, Brazil

## Abstract

The authors report and discuss the clinical and radiological features of a immunocompetent patient with chronic progressive monoarthritis tuberculosis of the hip without systemic symptoms such as fever, and weight loss presenting as caseating abscess and severe destruction of the hip joint, treated with resection arthroplasty.

## Introduction

Osteoarticular tuberculosis is a chronic inflammatory disease caused by *Mycobacterium tuberculosis* that affects joints. It reaches skeleton and joints by hematogenous spread. The clinical presentation includes multiple joint involvement with fever and weight loss, and periarticular abscess formation [1]. However, monoarthritis consisting localized pain for months followed by indolent progressive inflammation has been described in older population [2]. Failure to consider tuberculosis in the differential diagnosis of osteoarticular lesions in an individual from an endemic area of tuberculosis may substantially delay diagnosis.

We report and discuss the clinical and radiological features of a patient with chronic progressive monoarthritis tuberculosis of the hip presenting as caseating abscess and severe destruction of the hip joint, treated with resection arthroplasty.

## Case presentation

**Figure 1:**
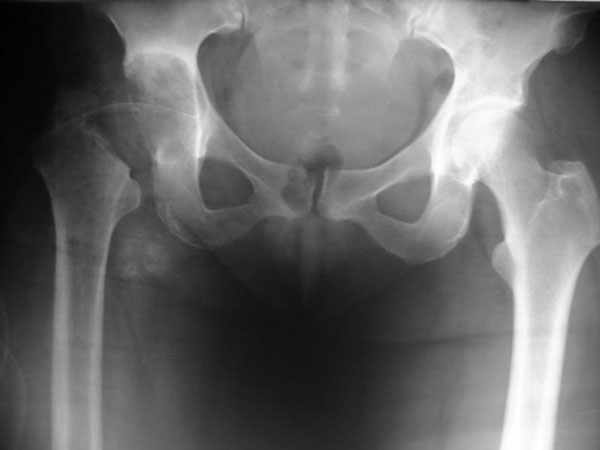
**Hip x-ray showing severe desctruction of periarticular bony destruction and deformity of the right hip**.

**Figure 2:**
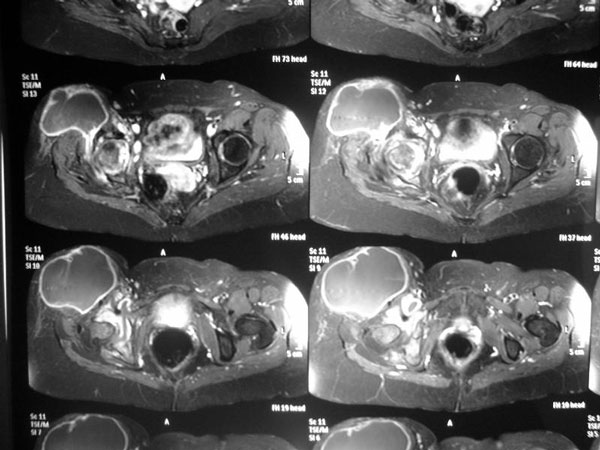
Magnetic resonance imaging of the hip showing a large periarticular abscess and destruction of the right femour head.

A 58-year-old female white brazilian patient presented with complaints of progressive mechanical pain in the right trochanteric area for 3 years that made it impossible to lean on the right side. Patient was in good general condition and denied systemic symptoms such as fever, weight loss, and loss appetite, and neither had any notion of primary tuberculosis in her past nor any evidence of the disease in her medical history. Routine laboratory tests were within normal limits. HIV-test was negative. Patient was seen multiple times by his primary care physician who prescribed non-steroidal anti-inflammatory drugs. Imaging studies showing severe desctruction of periarticular bony destruction and deformity of the right hip. Magnetic resonance imaging of the hip showing a large periarticular abscess and destruction of the right femour head (Figure 1 and 2). Needle aspiration of the abscess revealed *M. tuberculosis* on cultures and a tuberculin test done at this time was positive (21 mm). Antituberculous therapy (rifampicin, isoniazid, pyrazinamide) was introduced for 9 months after drainage of the abscess, and resection arthroplasty with osteotomy of the great trochanter. Histological examination of the trochanteric bone revealed an inflammatory reaction with a granuloma with caseous necrosis and giant Langerhans cells with numerous acid-fast bacilli. The clinical course was uneventful. No recurrence was observed after a follow-up of one year.

## Discussion

Osteoarticular tuberculosis has become very rare in western countries, but it is still a common problem in under developing countries [3]. After the spine, the hip joint is the most common site of involvement for tuberculosis, and constitutes approximately 15% of all cases of osteoarticular tuberculosis [4]. The common age of presentation is in the second and third decades. In Stages II and III of the disease, the radiologic features are very obvious and diagnostic, and almost always predict the final clinical outcome. A progressive pattern of destruction of the hip occurs in patients who are not treated. Treatment including drainage of collections, and tuberculostatic drugs must be instituted early with an aim of salvaging the hip. Like observed in our patient, the earliest manifestation of tuberculous arthritis is pain, which precede signs of inflammation for months and occasionally years. Imaging studies initially may show soft tissue swelling but later demonstrate osteopenia, periosteal thickning, periarticular bony destruction, and progressive destruction of cartilage and bone [5]. If the patient is not treated, cold abscesses and draining sinuses often develop.

Tuberculosis of the hip must be in the differential diagnosis of chronic pain of the hip including osteoarthritis, pseudogout, psoriatic arthritis and reumathoid arthritis. In additional to imaging studies, a detailed family and environmental history for pulmonary tuberculosis, history of tuberculosis vaccination, and a tuberculin skin test should be performed.

## Consent

Written informed consent was obtained from the patient for publication of this case report and accompanying images. A copy of the written consent is available for review by the Editor-in-Chief of this journal.

## Competing interests

The authors declare that they do not have any competing interests.

## Authors' contributions

CR and MA analyzed and interpreted the patient data regarding the diagnosis of tuberculosis and treatment. DF, RS and LG were major contributors in writing the manuscript. All authors read and approved the final manuscript.
